# Mapping and verification of grain shape QTLs based on an advanced backcross population in rice

**DOI:** 10.1371/journal.pone.0187553

**Published:** 2017-11-16

**Authors:** Duo Xia, Hao Zhou, Lei Qiu, Haichao Jiang, Qinglu Zhang, Guanjun Gao, Yuqing He

**Affiliations:** National Key Laboratory of Crop Genetic Improvement and National Centre of Plant Gene Research, Huazhong Agricultural University, Wuhan, China; Institute of Genetics and Developmental Biology Chinese Academy of Sciences, CHINA

## Abstract

Grain shape is a key factor that influences both the appearance quality and grain yield of rice. To clarify the genetic basis of grain shape, an advanced backcross population was developed from the cross of a slender *indica* variety Jin23B and a round *japonica* variety QingGuAi, and a total of 10 quantitative trait loci (QTLs) for grain shape were detected over 2 years. Three QTLs, *qGW1*, *qGS3* and *qGS7* have large effects on grain shape and were detected in both years. To further validate their effects, the BC_4_F_2_ populations of the three QTLs were constructed. The alleles from QingGuAi of *qGW1* and *qGS7* both greatly increase grain width and the alleles from Jin23B of *qGS3* greatly increase grain length. The allele variations of the three QTLs lead to the totally different grain shape of the two rice varieties. Our study provides insights into the genetic bases of grain shape and will promote the improvements of grain quality and yield in rice.

## Introduction

As one of the most important commercial crops, rice provides food for more than half of the world`s population. With the development of modern society, consumers not only concern about the yield but also the quality of rice. Grain shape, a major determinant of grain weight, plays a pivotal role in grain yield and appearance quality. Investigating the genetic basis of grain shape will be beneficial for improving both the yield and the quality of rice. Despite the fact that several grain shape genes have been identified, only a few loci have been employed in the rice breeding process[1–3].

Rice grain shape is determined by grain length, grain width and grain thickness. Till now, there are about a dozen of grain shape genes that have been cloned from natural varieties[1–8]. *GS3*, a major QTL that controls grain length and weight, was the first cloned gene for grain shape[9]. A C to A mutation in the second exon of *GS3* leads to a premature stop and results in an enhanced grain length. Researchers have found that *GS3* was selected during domestication and the A allele mutation was originated from the *Japonica* group and introgressed into the *Indica* gene pool[1, 10]. The *GW2*, a QTL for grain width identified by positional cloning, encodes a novel RING-type protein with E3 ubiquitin ligase activity[6]. *GW2* is a negative regulator of grain width and loss-of-function of *GW2* results in a wider grain. Another negative regulator of grain width is *GW5*, namely *GSE5*. *GW5*/*GSE5* encodes plasma membrane associated protein and the two types of deletion in the promoter region of *GW5*/*GSE5* caused a decreased expression of *GW5*/*GSE5* and an increased seed width[3, 11–13]. *GS5* encodes a putative serine carboxypeptidase and positively regulates grain size. Polymorphisms in the *GS5* promoter lead to different expression levels of *GS5* and results in the variation of grain size[5]. *GW8*, which encodes the transcription factor OsSPL16, is another positive regulator of grain size. A high expression level of *GW8* gives rise to an increased grain width[2]. The cloning and characterization of these grain shape genes has enriched our knowledge of how grain shape are regulated and encouraged marker-assisted breeding to target these genes in rice. However, most of those genes are with large effects, a great number of grain shape loci with minor effects have not been identified. The genetic bases of the morphological differences of grains have not been fully explored yet.

Quantitative Trait Loci (QTL) mapping is a powerful technique for dissecting the genetic basis of traits and species differences[14]. Most QTL mapping populations in rice are primary populations such as F_2_ populations, recombinant inbred lines (RILs), and thus QTLs with minor effect might not be detected due to the complicated background. In contrast, using advanced mapping populations which share a more uniform genetic background, can overcome this problem and increases the ability of detecting QTLs with small effects [15, 16].

In this study, we constructed a BC_3_F_1_ population containing 240 families using Jin23B(an *indica* accession with slender grain) as the recurrent parent and QingGuAi (a *japonica* accession with small round grains) as the donor parent. The BC_3_F_1_ population was planted for two years and for each year, grain length and width were measured. QTL analyses for grain length and width were performed based on this population and a total of 10 QTLs were detected, three of which were detected both in 2013 and 2014. Using three segregation population of BC_4_F_2_, we confirmed the effects of these three QTLs.

## Materials and methods

### Population development and trait evaluations

The population was developed using an *indica* accession Jin23B (slender grain) as the recurrent parent and a *japonica* accession QingGuAi (round grain) as the donor parent. We firstly cross QingGuAi with Jin23B to obtain F_1_ generation, and then we backcross the hybrids to Jin23B for three times and obtained 240 BC_3_F_1_ plants. The BC_3_F_2_ population contained 240 lines were derived from self-cross seed of every BC_3_F_1_ plant. Selected lines in the BC_3_F_1_ population were backcross to Jin23B to obtain BC_4_F_1_, and the self-cross seed of these BC_4_F_1_ plants were used to develop BC_4_F_2_ segregating population of each QTL. The BC_3_F_1_ population was planted in 2013, the BC_3_F_2_ population was planted in 2014, the BC_4_F_2_ segregating populations of *qGW1*, *qGS3* and *qGS7*was planted in 2015, and parents were planted in all three years, during the normal rice growing seasons (from mid-May to early October) at the experimental field of Huazhong Agricultural University in Wuhan, China. And during the growing seasons of 2013 in Wuhan, there was a sustained high temperature (above 36 centigrade) from late July to late August.

Harvested rice grains were air-dried and stored at room temperature for at least 3 months before testing. Fully filled grains for each plant were used for measuring grain length (mm) and grain width (mm). Ten randomly chosen grains from each plant were lined up length-wise along a vernier caliper to measure grain length, and then arranged by breadth to measure grain width.

### Genetic map construction and QTL analysis

According to the genetic linkage map reported by Qiu et al. [17], 105 SSR markers and 8 InDel markers evenly distributed over all 12 chromosomes were used to screen the 240 BC_3_F_1_ plants. The SSR assay was performed with 4% urea polyacrylamide gels migration and silver staining as reported by Panaud et al. [18]. A genetic linkage map was constructed using the Kosambi mapping function of MapMaker/Exp3.0 program[19]. QTL analysis was performed by composite interval mapping (CIM) method using WinQTLCart version2.5 software [20] with a logarithm of odds (LOD) threshold of3.0.

## Results

### Performance of the BC_3_F_1_ population

**Fig 1:**
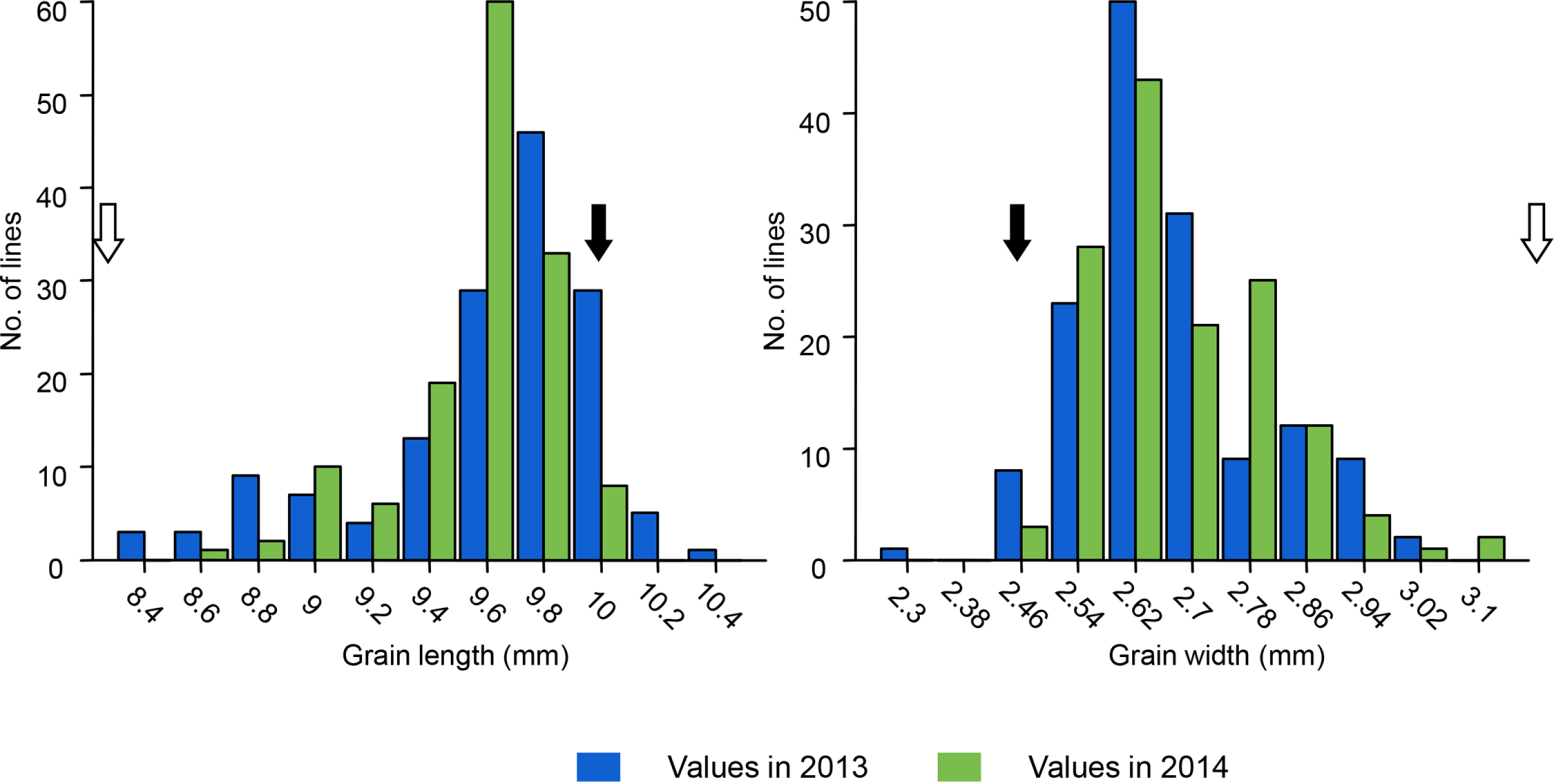
Frequency distribution of grain length and grain width of the BC_3_F_1_ population in 2013 and 2014. *Black arrows* and *white arrows* indicate the average values of Jin23B and QingGuAi, respectively.

The receptor parent Jin23B is an *indica* variety with slender grain (long 9.75mm and wide 2.47mm), and the donor parent QingGuAi is a *japonica* variety with round grain (long 7.97mm and wide 3.16mm) (Fig 1). The grain length showed a discontinuous variation and followed the bimodal distribution in both years. The grain width also showed a bimodal distribution and the two peaks were close to each other.

Table 1 shows the descriptive statistics of the two traits in two years, grain length in 2013 (GL13), grain width in 2013 (GW13), grain length in 2014 (GL14) and grain width in 2014 (GW14). Two years’ phenotypes show significant correlations with each other and grain length is negatively correlated with grain width in two years.

**Table 1:** Grain length and grain width of the BC_3_F_1_ population in years 2013 and 2014.

	GL13 (mm)	GW13 (mm)	GL14 (mm)	GW14 (mm)
Mean	9.72	2.71	9.67	2.73
SD	0.41	0.13	0.26	0.13
Min	8.48	2.37	8.76	2.53
Max	10.40	3.08	10.19	3.14
*Correlation*				
GL13 (mm)	1			
GW13 (mm)	-0.29***	1		
GL14 (mm)	0.62***	-0.49***	1	
GW14 (mm)	-0.56***	0.37***	-0.39***	1

GL13, grain length in 2013; GW13, grain width in 2013; GL14, grain length in 2014; GW14, grain width in 2014.

*** Significant at *P*< 0.001

### QTL mapping for grain shape

**Fig 2:**
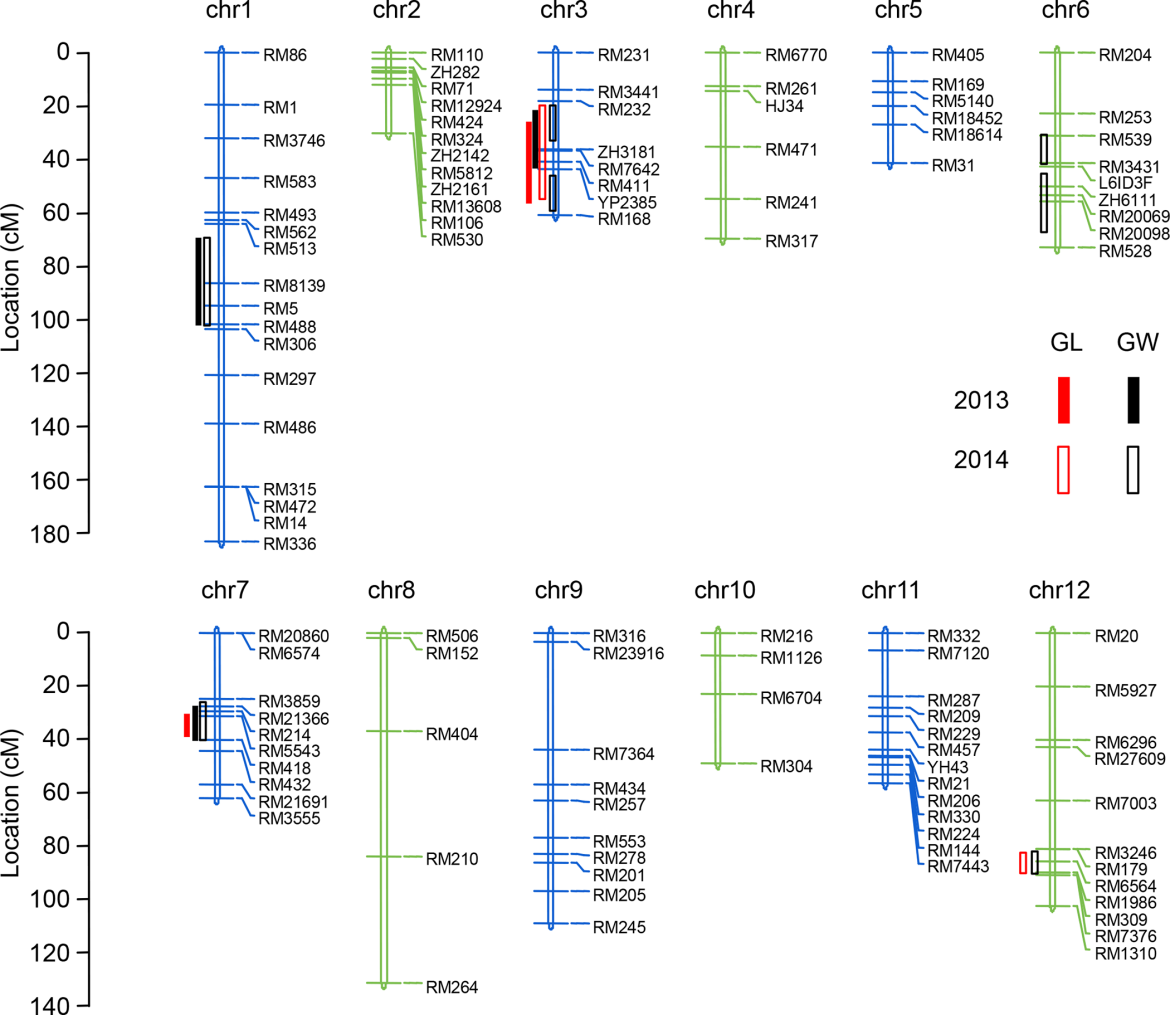
Distribution of putative QTLs for grain length and grain width on the linkage map. *Red and Black bars* indicate the QTLs for grain length and grain width, respectively. *Solid and Hollow bars* indicate the QTLs detected in 2013 and 2014, respectively. *chr*, chromosome. ‘ZH3181’, ‘YP2385’, L6ID3F and ‘ZH6111’ are InDel markers developed by our lab.

A total of 10 QTLs for grain shape were identified on chromosomes 1, 3, 6, 7 and 12 in both years (Table 2; Fig 2). The phenotypic variance explained by each QTL ranged from 3.62% to 32.38%.

**Table 2:** Putative QTLs for grain length and grain width detected in the BC_3_F_1_ population derived from Jin23B and QingGuAi two years.

QTL	Chromosome	Interval	Location (cM)	2013			2014		
				LOD	Add (mm)	PVE	LOD	Add (mm)	PVE
*qGL3*	3	RM232-RM168	47.51	16.79	0.85	24.99%	18.52	0.58	32.38%
*qGL7*	7	RM21366-RM5543	27.51	14.30	0.38	12.80%			
*qGL12*	12	RM179-RM7376	82.11				5.25	-0.09	8.14%
*qGW1*	1	RM513-RM306	83.11	16.08	-0.21	24.70%	11.23	-0.18	20.37%
*qGW3a*	3	RM232-RM411	32.91	4.71	-0.13	7.68%	5.81	-0.16	13.01%
*qGW3b*	3	YP2385-RM168	54.51				5.67	-0.16	13.11%
*qGW6a*	6	RM539-RM3431	33.41				5.18	-0.12	9.57%
*qGW6b*	6	L6ID3F-RM528	64.01				6.39	-0.17	15.05%
*qGW7*	7	RM3859-RM418	31.91	8.46	-0.11	10.12%	15.96	-0.18	28.56%
*qGW12*	12	RM3246-RM7376	88.51				4.67	0.04	7.31%

*Add*, the additive effect of each QTL; *PVE*, the phenotypic variance explained by each QTL; *LOD*, logarithm of odds; *qGL*, QTL for grain length; *qGW*, QTL for grain width

**Fig 3:**
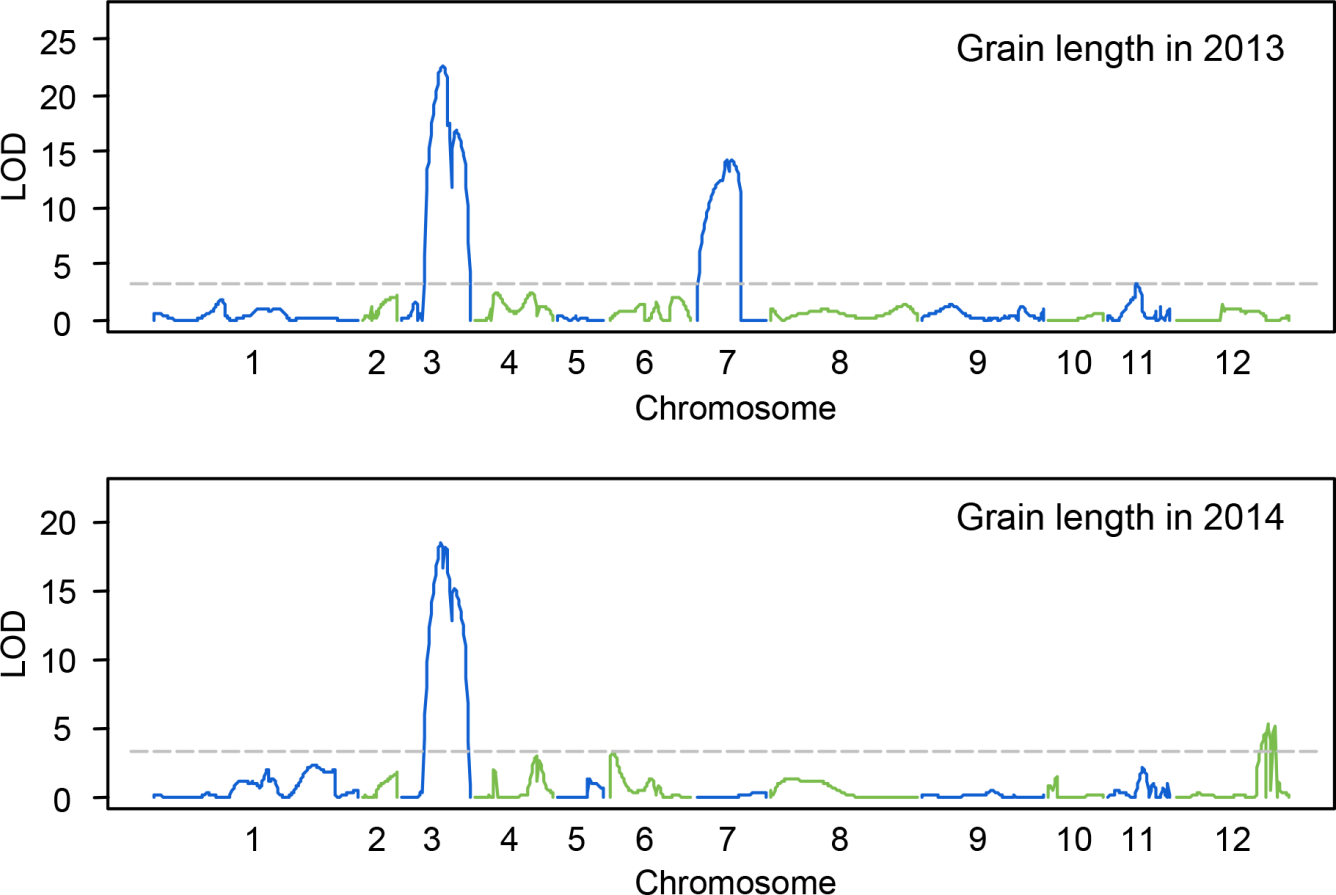
Graph for QTL mapping results of grain length of BC_3_F_1_ population in 2013 and 2014.

For grain length, three QTLs were distributed on chromosome 3, 7 and 12 (Fig 3). The QTL cluster *qGL3*, located between RM231and RM168 on chromosome 3, was detected in both years and explained 24.99% of phenotypic variation in 2013 and 32.38% of the phenotypic variation in 2014. A QTL, *qGL7* located betweenRM21366 and RM5543 on chromosome 7, was only detected in 2013 and explained 12.80% of phenotypic variation. Another QTL, *qGL12* located between RM179 and RM7376 on chromosome 12, was only detected in 2014 and explained 8.14% of phenotypic variation (Fig 3).

**Fig 4:**
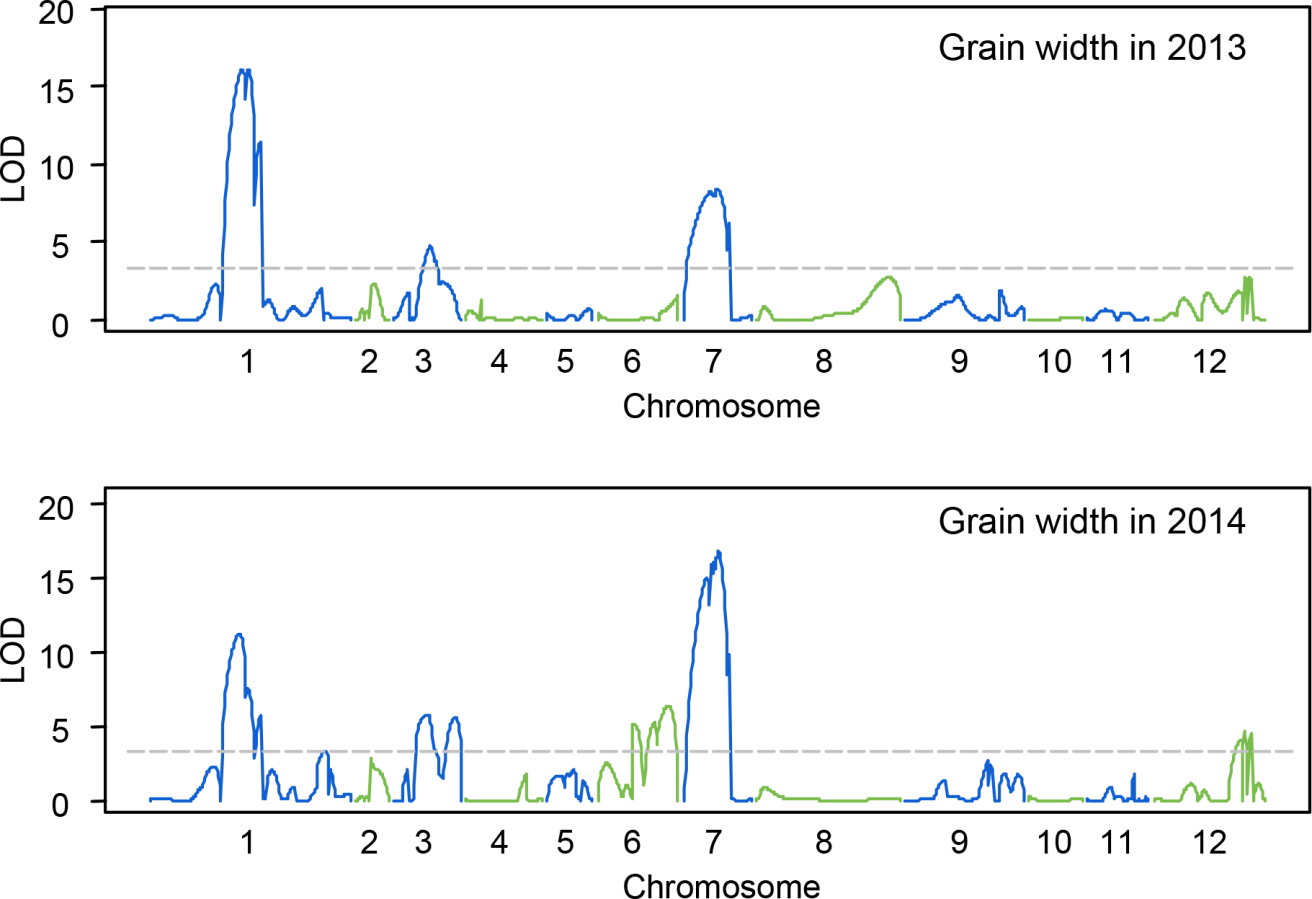
Graph for QTL mapping results of grain width of BC_3_F_1_ population in 2013 and 2014.

For grain width, seven QTLs were detected on chromosome 1, 3, 6, 7 and 12 (Fig 4). The QTL flanked by RM513 and RM306 on chromosome 1, *qGW1*, was detected in both years and explained 24.7% of the phenotypic variation in 2013 and 20.37% of the phenotypic variation in 2014. *qGW7*, a QTL flanked by RM3859 and RM418 on chromosome 7, was also detected in two years and explained 10.12% and 28.56% of the phenotypic variation, respectively. Two QTLs on chromosome 3, *qGW3a* and *qGW3b*, were located in adjacent regions flanked by RM231-RM411, and YP2385-RM168, respectively. *qGW3a* was detected in both years and accounted for 7.68% and 13.01% of the phenotypic variation, respectively. Whereas *qGW3b* was only detected in 2014 and accounted for 13.11% of the phenotypic variation. Another two QTLs on chromosome 6, *qGW6a* and *qGW6b*, were located in adjacent regions flanked by RM539 and RM3431, and L6ID3F and RM528, respectively. These two QTLs were only detected in 2014 and explained 9.57% and 15.05% of the phenotypic variation, respectively. The last QTL, *qGW12*, was only detected in 2014 and accounted for 7.31% of the phenotypic variation.

The region flanked by RM232 and RM168 on chromosome 3 and consisting of three QTLs, *qGL3*, *qGW3a* and *qGW3b*, and the region flanked by RM3859 and RM418 on chromosome 7 and consisting of two QTLs, *qGL7* and *GW7*, were both responsible for grain length and grain width, and were termed *qGS3* and *qGS7*, respectively, hereafter.

### Validate the genetic effect of *qGW1*, *qGS3* and *qGS7*

**Fig 5:**
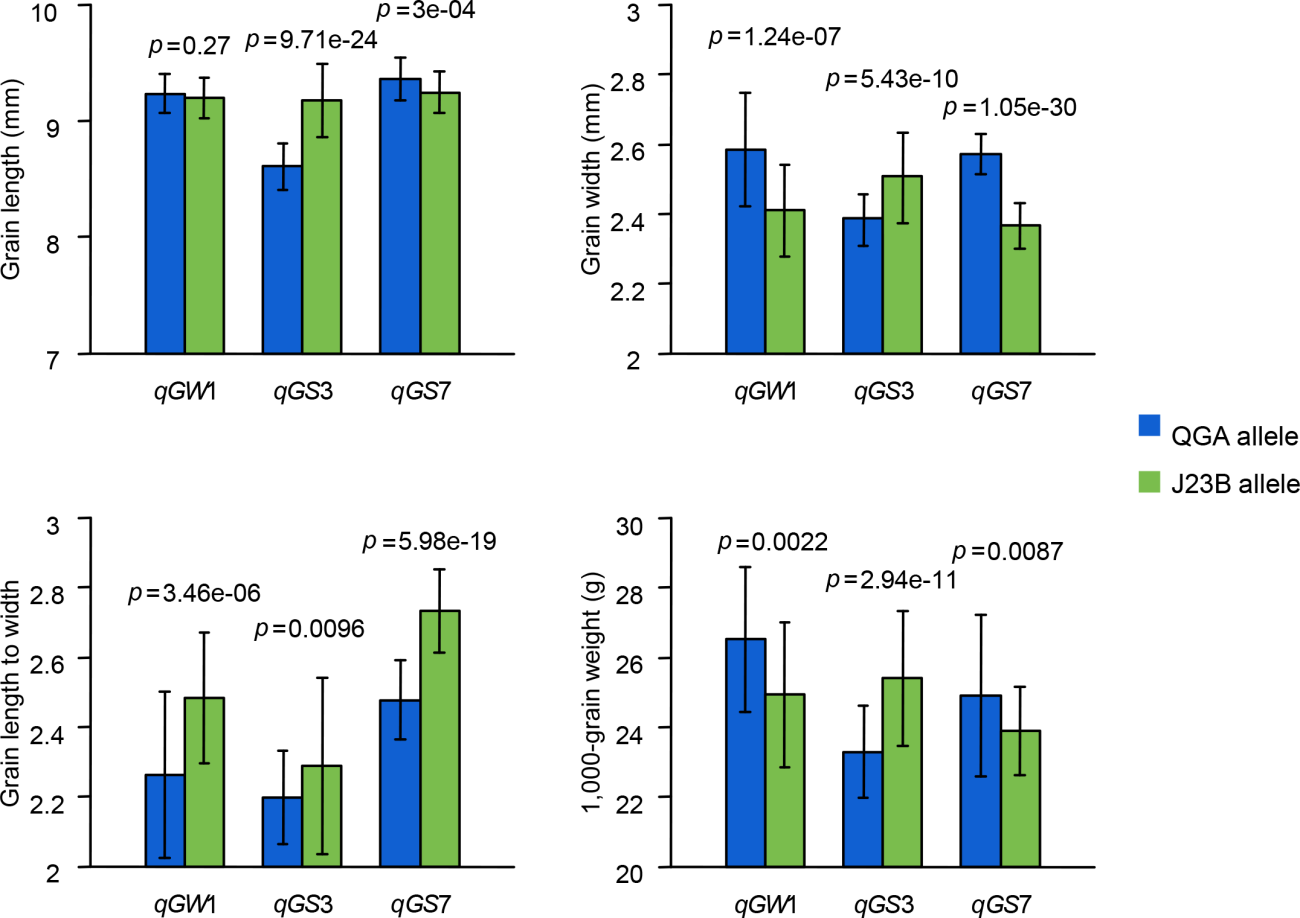
Genetic effects of *qGW1*, *qGS3* and *qGS7* on grain length, grain width, grain length to width and 1,000-grain weight. *Blue bar* represent alleles from QingGuAi (QGA), *Green bar* represent alleles from Jin23B (J23B). *P*-value based on two-way *t-test*. Error bars are based on standard deviation of each genotype.

The BC_4_F_2_ segregation populations of *qGW1*, *qGS3* and *qGS7* were used to confirm the genetic effect of these QTLs. The *qGW1* locus from QingGuAi increased grain width by 0.18 mm and had no effect on grain length (Fig 5). The *qGS3* locus from Jin23B increased both grain length and grain width by 0.57 mm and 0.13 mm, respectively (Fig 5). The *qGS7* locus from QingGuAi increased both grain length and grain width by 0.11 mm and 0.21 mm, respectively (Fig 5). And all three QTLs have significant effects on grain length to width and 1,000 grain weight (Fig 5).

## Discussion

In the present study, we totally detected 10 QTLs in two years for grain shape (Table 2). Among them, a QTL cluster on chromosome 3 possessed a major QTL for grain length and a minor QTL for grain width and was detected in both two years. When we further compared the position of this cluster to previous studies, we found that it contained both the *GS3* and *GL3*.*1*, which are two cloned grain shape genes and *GS3* is a major QTL for grain length[21, 22]. The *GS3* allele exerts a strong effect on grain length and grain weight and a slight influence on grain width [21], and a C to A mutation induces longer grain length [1]. A single amino acid diversity (D364E) of *GL3*.*1* refers to the variation of grain length and does not show a significant effect on grain width[22]. In this study, sequencing of the *GS3* region showed that diversity exists in Jin23B and QingGuAi on the functional SNP (C165A), and Jin23B with an A-allele displays a phenotype of longer grain length, which was consistent with Noriko's results. And sequencing of the coding region of *GL3*,*1* showed that the functional SNP was identical in Jin23B and QingGuAi and the 364 amino acid was both D (Asp), which suggesting that *GL3*.*1* may not be responsible for the *qGL3* region[23]. These results suggest that the *GS3* allele might be a candidate gene for *qGL3*. What is more, we detected a grain width QTL *qGW3a* in this region, and the effect of it was consistent with the effect of *GS3* on grain width, providing an evidence for the hypothesis that *GS3* was a candidate gene of *qGL3* and *qGW3a*.

*GW5* is the major gene conferring grain width and grain weight in rice. The different genotypes of *GW5* lead to the slender grain in *indica* and round grain in *japonica*. The parents of our mapping population, Jin23B and QingGuAi, are respectively an *indica* with slender grain and a *japonica* with round grain. However, *GW5* did not contribute to the grain width difference between the two parents which both have a functional *GW5* allele. Instead, two grain width QTLs, *qGW1* and *qGW7*, derived from QingGuAi, increased grain width by 0.18 mm and 0.21 mm, respectively (Table 2 and Fig 5). This indicates that there are lots of QTLs contribute to the grain width difference between *indica* and *japonica* and these QTLs have donor effects on grain width.

Advanced backcross QTL analysis (AB-QTL analysis) was proposed as a molecular-breeding method that integrates QTL analysis with germplasm development in crosses between adapted and wild germplasm [15].The efficiency of using the advanced backcross (AB) method to identify QTL is well-documented [15, 24–26]. We could detect QTLs using advanced backcross population and in the meanwhile, we could introduce new genes into Jin23B to improve the grain shape (Table 2 and Fig 5). One of the advantages of the advanced backcross methodology is the relative ease of then creating near-isogenic lines (NILs) to further test the identified QTL. This is useful not only for introducing new traits into cultivated varieties but also for further characterizing or fine mapping the QTL. The success in creating lines with improved characteristics using the NIL strategy has been variable, with greater success having been reported for agronomic traits such as disease resistance, yield and fruit shape[27, 28]. In this study, we constructed a BC_3_F_1_ population to detect QTLs for grain shape and we used BC_4_F_2_ lines as NILs to test the identified QTLs. Compared with other methodologies, using advanced backcross is time-easing. Besides, we could see that by introducing *qGW1* and *qGS7* into Jin23B, not only grain width is increased but also the 1000-grain weight (Fig 5), this indicates that these QTLs may have a potential to increase rice yield through increasing grain weight. Once the effects of these QTLs were verified, the population could be used to fine mapping these QTLs. What should be mentioned is that using advanced backcross population still has some limitations in estimating the genetic effect of a QTL. Furthermore, NIL studies have been shown to be very useful in expanding our knowledge of gene action, evolutionary implications, and developmental pathways [29–32]. Thus it seems highly likely that with further research, the QTLs identified in this study could be used to improve not only the grain shape of rice but also our knowledge of the regulatory network of grain shape.
